# Acceptance and Commitment Therapy for anxious children and adolescents: study protocol for a randomized controlled trial

**DOI:** 10.1186/1745-6215-14-140

**Published:** 2013-05-15

**Authors:** Jessica Swain, Karen Hancock, Angela Dixon, Siew Koo, Jenny Bowman

**Affiliations:** 1 School of Psychology, The University of Newcastle, Newcastle, NSW 2308, Australia; 2 Department of Psychological Medicine, The Children’s Hospital at Westmead, Sydney, NSW 2145, Australia

**Keywords:** Acceptance and Commitment Therapy (ACT), Anxiety, Anxiety disorders, Adolescents, Children, Cognitive Behavior Therapy (CBT), Randomized Controlled Trial (RCT)

## Abstract

**Background:**

Anxiety disorders affect approximately 10% to 20% of young people, can be enduring if left untreated, and have been associated with psychopathology in later life. Despite this, there is a paucity of empirical research to assist clinicians in determining appropriate treatment options. We describe a protocol for a randomized controlled trial in which we will examine the effectiveness of a group-based Acceptance and Commitment Therapy program for children and adolescents with a primary diagnosis of anxiety disorder. For the adolescent participants we will also evaluate the elements of the intervention that act as mechanisms for change.

**Methods/design:**

We will recruit 150 young people (90 children and 60 adolescents) diagnosed with an anxiety disorder and their parent or caregiver. After completion of baseline assessment, participants will be randomized to one of three conditions (Acceptance and Commitment Therapy, Cognitive Behavior Therapy or waitlist control). Those in the Acceptance and Commitment Therapy and Cognitive Behavior Therapy groups will receive 10 × 1.5 hour weekly group-therapy sessions using a manualized treatment program, in accordance with the relevant therapy, to be delivered by psychologists. Controls will receive the Cognitive Behavior Therapy program after 10 weeks waitlisted. Repeated measures will be taken immediately post-therapy and at three months after therapy cessation.

**Discussion:**

To the best of our knowledge, this study will be the largest trial of Acceptance and Commitment Therapy in the treatment of children and young people to date. It will provide comprehensive data on the use of Acceptance and Commitment Therapy for anxiety disorders and will offer evidence for mechanisms involved in the process of change. Furthermore, additional data will be obtained for the use of Cognitive Behavior Therapy in this population and this research will illustrate the comparative effectiveness of these two interventions, which are currently implemented widely in contemporary clinical practice. Anticipated difficulties for the trial are the recruitment and retention of participants, particularly adolescents. To avert these concerns and maximize recruitment, several strategies will be adopted to optimize referral rates as well as reduce participant drop-outs.

**Trial registration:**

This trial is registered with the Australian and New Zealand Clinical Trials Registry, registration number: ACTRN12611001280998

## Background

With a prevalence rate of 10% to 20%, anxiety disorders are the most common mental health concern affecting children and adolescents [1,2]. Young people with anxiety are typically underrepresented in clinical research, and anxiety in children is often minimized by health professionals, potentially due to a common perception that in this population anxiety is developmental, transient and innocuous [3,4]. Despite this, anxiety in childhood increases the likelihood of academic and social skills difficulties as well as substance abuse, and is often enduring if untreated [2]. Furthermore, a childhood history of anxiety is a common precursor to depression, and has been found to predict anxiety and depression in later life [5-7].

In a recent review of the best available evidence for the treatment of psychological disorders, Cognitive Behavior Therapy (CBT) was found to be the first-line evidence-based psychosocial intervention for anxiety among adults and is currently the most empirically supported therapeutic approach for children and adolescents [8]. In part, this is a consequence of insufficient evidence for alternative interventions [8], rather than findings indicating other treatments are unsuitable. Indeed, the dearth of population-specific research in this area is highlighted by the aforementioned review, which found a complete absence of studies assessing the efficacy of CBT in the treatment of panic disorder among children and variable levels of evidence for its use in other anxiety disorders in this population [8]. Furthermore, others have found that one in four children do not benefit from CBT [9]. As such, it is important that other interventions are developed and evaluated to address this shortcoming.

Acceptance and Commitment Therapy (ACT) has sparked increased interest among clinicians and researchers in the last decade [10]. ACT considers the fundamental cause of psychopathology and human suffering to be the interrelationships of cognition, language and life circumstances that lead to decreased capacity to modify or continue exhibiting behaviors that are in the service of personal values [11]. ACT aims to increase psychological flexibility; ‘the process of contacting the present moment fully as a conscious human being and persisting or changing behaviour in the service of chosen values’ [12]. Whereas other therapies focus on altering the content, frequency and form of private experience (thoughts, feelings and sensations), ACT works to modify the function of internal experience - such as supporting individuals to recognize thoughts for what they are, simply thoughts and not necessarily the truth - and thus reduce their bearing on behavior [13]. ACT focuses on assisting clients to live valued meaningful lives [11]. To do this, six core therapeutic processes organized in a ‘hexaflex’ model are employed, including ‘acceptance,’ ‘defusion,’ ‘values,’ ‘committed action,’ ‘the present moment’ and ‘self-as-context’ [14]. These processes are interrelated and support each other in increasing psychological flexibility.

ACT has a growing empirical base demonstrating its efficacy for an array of problems, including the treatment of anxiety concerns among adults such as social phobia [15,16], generalized anxiety disorder [17] and mathematics anxiety [18]. Indeed, in the first known review of published ACT controlled trials up to 2005, the authors found ACT to be superior to control conditions, waitlists and treatment as usual at both post-intervention and at follow-up across a myriad of different problems from psychosis to work stress [12]. Whilst evidence for the use of ACT in adult populations with anxiety has grown, there is currently a paucity of research examining the efficacy of ACT in children and adolescents with anxiety. A literature search produced only one published study, that being a case study [19]. However, preliminary research evidence supports the use of ACT among young people with other problems including depression [20], anorexia [21], chronic pain [22] and high risk sexual behavior [23].

Research evidence has supported the use of mindfulness, one of the ACT core processes, in the treatment of young people. Four studies have assessed the impact of mindfulness-based stress reduction among children and/or adolescents with anxiety and found it to be effective in reducing anxious symptoms [24-27]. A review of these studies has previously been conducted [28]. Although these studies show some early support for the use of ACT for the treatment of childhood problems including anxiety, they are subject to several methodological issues - small samples, a lack of either control group or random assignment, few objective measures, potential biases from recruited volunteers, reliance on self or non-blind parent or teacher reports and employment of non-clinical samples, and/or the inclusion of only one component of the ACT model (that is, mindfulness) - that limit their validity. More rigorous research is required to solidify the effectiveness of mindfulness in the treatment of child anxiety disorders and to extend the research into other ACT core processes.

Clinical research has typically focused on assessing the efficacy of interventions. However, this approach does not assist in the identification of the specific techniques that are empirically effective or, conversely, those that are harmful [14]. Identification of the mechanisms of action within a specific treatment could support clinical practice and enable interventions to be tailored to meet individual client needs. It has been proposed that ACT works by supporting increased acceptance of internal experience and reducing fusion with negative thoughts to enable valued living, referred to in ACT as increasing psychological flexibility [12]. ACT studies have typically focused on evaluation of the core processes of the model, as described above, to examine the validity and impact of each [12]. These studies have found support for the roles of defusion [29] and acceptance [17,30], and some support for the role of values and/or committed action [17] in reducing psychopathology. Ultimately, this emerging research has led to the genesis of three ACT mediational hypotheses, that psychological inflexibility precedes suffering among clinical and non-clinical populations; ACT increases psychological flexibility; and psychological flexibility leads to enhanced well-being, decreased clinical symptoms and increased value-based activities [14]. However, these studies are preliminary and subject to several methodological limitations including measures that lack psychometric evaluation, the use of purely self-report measures and the use of measures that concentrate on a small number of core processes, leaving other parts of the ACT model untested [31]. Thus, to build upon the current empirical literature in this area, this randomized controlled trial design includes the investigation of mechanisms of change in adolescents with anxiety.

In summary, preliminary investigations of ACT in the treatment of adult anxiety have produced promising results. Other studies have also supported the use of mindfulness - one of the six ACT core processes - in the treatment of childhood anxiety, which suggest that approaches employed within ACT appear to be suitable for child populations. To the best of our knowledge, this will be the first randomized controlled trial to examine the effectiveness of ACT in young people with a diagnosed anxiety disorder. Given the popularity and use of ACT in clinical practice [10], it is imperative that this form of intervention be empirically evaluated for its efficacy. Thus, the aim of this research is to examine the effectiveness of a manualized ACT group-therapy program in the treatment of anxiety disorders among children and adolescents. It is hypothesized that ACT will be at least as effective in the treatment of anxiety disorders in these populations relative to a manualized CBT group-therapy program, and that ACT will be more effective in the treatment of anxiety disorders in this population relative to the control condition at both immediate post-treatment and at three-month follow-up on outcome measures. The secondary aim of the trial concerns the adolescent participants and is to identify the mechanisms of change surrounding the intervention that are critical to changes in outcome measures. It is hypothesized that these will include decreased experiential avoidance and cognitive fusion, as well as increased emotional awareness, acceptance and valued living.

## Methods

### Study design

**Figure 1:**
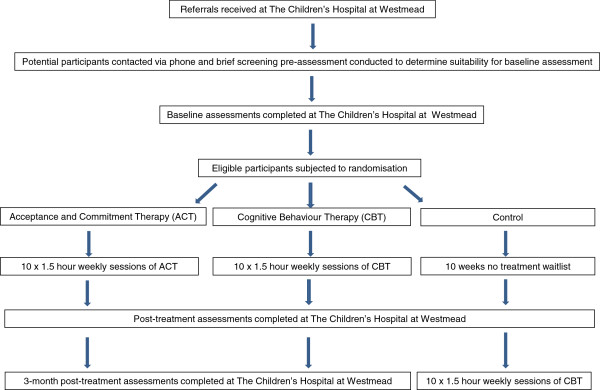
Study design.

This is a prospective randomized controlled trial. It is a three (group: two intervention and one control) by three (time: pre-, immediate post- and three-month post-treatment) repeated measures factorial design. The overall study design is illustrated in Figure 1.

### Participants

Participants will be approximately 150 children (90 younger and 60 older children) aged 7 to 17 years with a primary diagnosis of a Diagnostic and Statistical Manual of Mental Disorders (DSM-IV) anxiety disorder. The participants will be age-classified as ‘children’, aged 7 to 11 years, and ‘adolescents’, aged 12 to 17 years. Twelve years of age was determined as the cut-off point to be consistent with other research involving ACT as a treatment for problems among adolescents [20].

Participants will be recruited via referrals to the Department of Psychological Medicine, The Children’s Hospital at Westmead (CHW), Sydney, NSW, Australia. Referrals will be accepted from anyone, including health professionals (general practitioners, psychologists, pediatricians), educational providers, and self-referrals. Written informed consent will be obtained from the parent or caregiver of the children and from the child/adolescent. Ethical approval was obtained for this study from the Human Research Ethics Committee at CHW and at The University of Newcastle.

The researchers predict that the majority of referrals will come from school counselors, followed by word of mouth, then via parents’ referral through the CHW intake phone line. In terms of proportions, it is expected that 60% to 70% of referrals will come from school counselors. On the basis of recruitment efforts to date and requests to intake phone line, the researchers consider that the anticipated sample will be achieved.

### Inclusion criteria

1. Aged between 7 and 17 years

2. Criteria met for a primary diagnosis of a DSM-IV anxiety disorder (including panic disorder and/or agoraphobia, obsessive compulsive disorder, specific phobia, social anxiety disorder or generalized anxiety disorder)

3. Available and able to attend CHW for pre-treatment, immediate post-treatment and three-month post-treatment assessments as well as attending a minimum of 80% of therapy sessions

4. Have a parent or caregiver who is willing to attend and participate in the assessment as well as a minimum of 80% of therapy sessions.

### Exclusion criteria

1. Developmental or language delay, as reported by the parent or caregiver

2. Non-English speaker

3. Complex mental health problems such as psychosis, conduct disorder or active suicidality

4. Complex medical conditions with a high degree of medical dependence that would prevent them from being able to attend at least 80% of sessions

5. Attention deficit disorder with hyperactivity (ADHD) that is not well controlled. In addition to initial screening via parent and teacher reports as well as by a physician, as appropriate, ADHD will be assessed by clinicians according to DSM-IV criteria during the initial assessment. It was determined that, unless ADHD is sufficiently controlled, the group-based 1.5 hour program is an unsuitable structure for both the child with this identified concern as well as other group members

6. Medicated with an anxiolytic or antidepressant for less than two months. However, in the instance of a participant experiencing a marked increase in symptom severity across treatment, there may be a need to consider pharmacotherapy. Participants will have weekly contact with the researchers across the course of the program and changes in functioning or status will be monitored in an ongoing way. Where a participant’s progress appears to be worsening, a case-by-case consideration of the need for a medical assessment for potential commencement of pharmacotherapy will be undertaken. In addition to this, the uptake of medication external to that identified across the trial will also be assessed at follow-up assessment. If a participant does utilize medication of this kind throughout the course of the study, they will be excluded from data analysis.

7. Post-traumatic stress disorder (due to the potential distress caused to other participants in the group and the specialized treatment required for this disorder)

8. Completed <70% of sessions or dropped out of treatment - those who become lost to follow-up will be placed in the intention-to-treat category.

### Procedure

#### Initial assessment

Following referral, the caregivers of potential child and adolescent participants will be briefly screened over the phone to determine suitability for baseline assessment using a checklist developed for this study. Information collected as part of this assessment will include psychiatric diagnoses and psychiatric symptoms - to identify anxiety as the primary presenting problem and consider differential diagnoses - as well as current and previous treatment including medication (type, dosage, period of pharmacological treatment) in accordance with the aforementioned inclusion and exclusion criteria. If deemed suitable at this point, they will be sent an information pack about the study including consent forms, and a battery of questionnaires to be completed by both the child/adolescent and caregiver as part of the baseline assessment. All assessment tools employed have demonstrated reliability and validity (described in detail below). To complete the baseline assessment the child/adolescent and parent will attend CHW to undertake a face-to-face diagnostic interview to determine the presence of an anxiety disorder using the Anxiety Disorders Interview Schedule for DSM-IV [ADIS-IV] (Silverman & Albano, 1996). ADIS-IV interviews of both parent/caregiver and child/adolescent which will be conducted separately by psychologists trained in the administration of the instrument. All interviews will be recorded for reliability purposes. The researchers conducting baseline assessments will be blinded to the treatment type to reduce potential.

#### Randomization

Following baseline assessment, eligible participants and their caregivers will be randomized to one of three conditions - ACT, CBT or a waitlist control group - for a period of 10 weeks (described in detail below). Each group will comprise up to eight children/adolescents as well as their caregivers. The researchers involved in this study are six registered psychologists, all trained in ACT and CBT. This will be a block randomized controlled trial, with the participant serving as the unit of randomization. Randomization will be undertaken via a publically available random assignment software application, ‘Graphpad’ [http://www.graphpad.com/quickcalcs/].

#### Follow-up assessment

Follow-up repeated measures assessment will be completed immediately post-treatment - or after 10 weeks for the control group - and three months post-treatment for both intervention groups. Following completion of the 10-week post assessment, the control group will complete a program of 10 × 1.5 hour sessions of CBT. Although it will not be possible to ensure the researchers conducting post-intervention and follow-up assessments will be blinded to the treatment type, to reduce potential bias these interviews will be recorded for reliability purposes and reviewed and re-rated by an independent assessor - a psychologist within the Department of Psychological Medicine with training in the use of the ADIS-IV - blind to the diagnosis obtained. Participants will also be assessed on the uptake of pharmacotherapy throughout the course of the trial at follow-up.

#### Outcome measures

### Primary outcome measures

*Anxiety Disorders Interview Schedule (ADIS-IV)*[32]*.* The ADIS-IV is a structured diagnostic interview that assesses for a range of DSM-IV disorders typically first diagnosed in childhood or adolescence [33] from the perspective of both child (ADIS-C) and parent (ADIS-P) [34]. The ADIS-C and ADIS-P demonstrate good-to-excellent clinician inter-rater agreement, diagnostic reliability and test-retest reliability [35,36].

*Multidimensional Anxiety Scale for Children (MASC)*[37]*.* The MASC is a 39-item self-report inventory of anxiety symptoms. It assesses four factors of anxiety including physiological symptoms, avoidance, social and separation anxiety [34,38]. Research has shown the MASC exhibits acceptable convergent and divergent validity, moderate-to-strong internal reliability, and adequate test-retest and discriminate validity [34,39,40].

*Child Behavior Checklist - Parent Form (CBCL)*[41]*.* The CBCL is a widely utilized standardized measure of children’s and adolescents’ (aged 5 to 18 years) emotional and behavioral functioning as well as social competence [42]. The social competence scales examine the child’s adaptive functioning including their activities, and social and school performance [42]. Behavioral and emotional functioning is assessed by 118 items, which describe an array of problems that children might experience [42,43]. Validity and reliability data were obtained in a sample of over 5,000 children and were found to be moderate-to-high and high, respectively [44].

For the primary aim of the trial, relating to efficacy of the intervention, the primary outcome measures will be whether the participant meets criteria for one or more DSM-IV anxiety disorders and/or clinically significant changes in severity scores on the ADIS-IV, CBCL or MASC assessments. Clinically significant change will be defined as a change in score that places the participant within a different severity range on the relevant assessment. For the ADIS-IV, this will be a shift in interference ratings between ‘Very severe’ (8), ‘Severe’ (6 to 7), ‘Moderate’ (4 to 5), ‘Mild’ (1 to 3) or ‘Absent’ (0) ranges [45]. For the CBCL, this will be represented by a change in t-scores among the ‘Clinical’ (≥69); ‘Borderline clinical’ (56 to 69) and ‘Normal’ (≤55) ranges [41]. For the MASC, in accordance with March [37], this will be a shift in t-score between the ‘Severe’ (≥70); ‘Moderate’ (56 to 69) and ‘Non-clinical’ (≤55) ranges. Secondary outcome measures will include anxiety symptoms; (depression symptoms; and quality of life and self-efficacy (Table 1).

**Table 1:** Primary and secondary outcome measures as they relate to the efficacy aim

**Intervention efficacy outcome measures**	**Assessment tool/measured factor**
*Primary measures*	
DSM-IV Anxiety disorder	ADIS-IV
Clinically significant change in anxiety severity	ADIS-IV, MASC, CBCL
*Secondary measures*	
Depression symptoms	CDI, CBCL
Quality of life and self-efficacy	CALIS-C
Demographic factors	FAD, age, sex

ADIS-IV, Anxiety Disorders Interview Schedule; CALIS-C, Children’s Anxiety Life Interference Scale - Child Form; CBCL, Child Behavior Checklist - Parent Form; CDI, Child Depression Inventory; DSM-IV, Diagnostic and Statistical Manual of Mental Disorders; FAD, Family Assessment Device; MASC, Multidimensional Anxiety Scale for Children.

### Secondary measures

*Child Depression Inventory (CDI)*[46]*.* The CDI is one of the most widely utilized and cited diagnostic instruments for depression in children [47,48]. It is a 27-item self-report assessment, adapted from the Beck Depression Inventory [48]. Research has found the CDI test-retest reliability to be moderate range for clinical samples and adequate internal consistency, concurrent validity [47] and discriminant validity have been established [46].

*Children’s Anxiety Life Interference Scale - Child Form (CALIS-C)*[49]*- Adolescents only.* The CALIS-C is a 10-item self-report questionnaire about the impact of fears and worries on an adolescent’s quality of life, self-efficacy and well-being [49]. Reliability estimates were found to be adequate; moderate-to-strong convergent validity and discriminant validity have been observed [49]. The CALIS-C also demonstrates sensitivity to change [49].

*McMaster Family Assessment Device (FAD)*[50]*.* The FAD is a 53-item inventory completed by caregivers on the structure, organization and patterns of transactions within families [50]. Six dimensions of family functioning are identified in the model including Problem Solving, Communication, Roles, Affective Responsiveness, Affective Involvement and Behavioral Control [50]. Moderate-to-strong reliabilities have been obtained for the FAD [50]. It also has established discriminant [50] and concurrent validity [51].

### Process measures – Adolescents only

*Avoidance & Fusion Questionnaire - Youth (AFQ-Y)*[52]*.* The AFQ-Y is a 17-item self-report measure of cognitive fusion and experiential avoidance for children and adolescents [52]. Confirmatory factor analysis has supported the hypothesized one-factor model of the AFQ-Y and internal consistency reliability was also found to be strong [52].

*Child and Adolescent Mindfulness Measure (CAMM-20)*[53]*.* The CAMM-20 is a 20-item self-report questionnaire that focuses on internal and external awareness as well as mindfulness [53]. Exploratory factor analysis found support for a CAMM-20 two-factor model of ‘Observing’, noticing and attending to stimuli including internal and external phenomena, and ‘Acting with Awareness (AWA)’ including items that involve absolute focus and engagement with activity in the present moment [53]. The internal consistency of both scales was in the moderate range [53].

*Positive and Negative Affect Schedule (PANAS-X)*[54]*.* The PANAS-X is a 20-item measure of emotional experience across two scales, Positive Affect (PA) and Negative Affect (NA) [55]. Respondents rate the extent to which they have experienced an emotion over a prescribed time [55]. Internal consistency reliabilities were found to be in the moderate-to-high range across both scales, and low correlations between the NA and PA scales indicate good discriminant validity [54], with similar results obtained in a sample of adolescents [53]. The PANAS-X demonstrates adequate construct validity and high internal consistency [54].

*Toronto Alexithymia Scale (TAS-20)*[56]*.* The TAS-20 is a 20-item self-report inventory of the construct of alexithymia and produces scores in three related domains: difficulty identifying feelings, difficulty describing feelings and externally-orientated thinking [57]. The TAS-20 has been found to demonstrate adequate convergent, discriminant and concurrent validity [58].

*Valued Living Questionnaire (VLQ)*[59]*.* The VLQ measures valued living or the degree to which an individual accesses their chosen values in everyday life [59]. The VLQ is comprised of two 10-item scales, where participants rate the importance of different domains of life - family, intimate relationships, parenting, friendship, work, education, recreation, spirituality, citizenship and physical self-care - and subsequently rate the consistency with which they have acted in accordance with their values in the past week [59]. Wilson *et al*. [59] found the VLQ to demonstrate adequate-to-good internal consistency across domains.

For the secondary aim of the trial, relating to the mechanisms for change, process outcome measures have been developed for adolescents only and have been selected in accordance with the domains hypothesized to be associated with treatment efficacy in line with results of previous research (described above). As such, primary process outcome measures include experiential avoidance, cognitive fusion, acceptance and valued living (Table 2).

**Table 2:** Primary outcome measures as they relate to the mechanism of change aim

**Mechanisms of change outcome measures**	**Assessment tool/measured factor**
Experiential avoidance	AFQ-Y, PANAS-X, TAS-20
Cognitive fusion	AFQ-Y
Acceptance	CAMM-20
Valued living	VLQ

AFQ-Y, Avoidance and Fusion Questionnaire - Youth; CAMM-20, Child and Adolescent Mindfulness Measure; PANAS-X, Positive and Negative Affect Schedule; TAS-20, Toronto Alexithymia Scale; VLQ, Valued Living Questionnaire.

#### Intervention

Participants allocated to ACT or CBT groups will complete a group-based therapy program of 10 × 1.5 hour sessions of the applicable treatment at CHW, which will be provided at no cost. Up to eight children or adolescents will be involved in each group program. Treatment will be conducted by between two and four psychologists, dependent upon final group numbers. All psychologists involved in the delivery of the intervention have been equally trained in both ACT and CBT, with the exception of one who has advanced training in CBT and intermediate training in ACT.

Caregivers will be involved concurrently in a “parent-as-coach” manner. Across both programs, the treatment will incorporate aspects that involve the participants and caregivers working independently of one another, as well as aspects that require working together. Both programs will also require regular completion of between-session practice tasks by both caregiver and the participant that will be reviewed at the subsequent session.

Both ACT [60-62] and CBT [63-65] programs will be based upon treatment manuals, tailored to the needs of participants. Manuals have been developed for children, adolescents, caregivers and therapists. Each program incorporates a series of psychological techniques consistent with the therapeutic modality employed. However, there are some commonalities across both interventions including psychoeducation, skills training and exposure. Although exposure to feared situations is a prominent technique employed across both ACT and CBT, the approach differs across therapies. In CBT, the focus is on challenging maladaptive thinking to enable performance of the exposure behavior, whereas in ACT, children or adolescents are encouraged to attempt to alter the relationship they have with their anxiety by distancing themselves from it and increasing their willingness to experience it.

### CBT program - Cool Kids (ages 7 to 11 years) and Chilled® (ages 12 to 17 years)

The CBT program will comprise the Cool Kids and Chilled® Programs developed at the Centre for Emotional Health, Macquarie University [66]. The effectiveness of Cool Kids and Chilled® in treating anxiety has been empirically demonstrated [66]. Cool Kids and Chilled® assist children and adolescents, respectively, to learn skills to recognize their emotions and combat anxiety, encouraging brave behavior and gradual engagement with feared situations. Because CBT is currently the most empirically supported therapeutic approach for children and adolescents [8], it was determined to be the most stringent comparison condition to employ within this trial. The ‘worry wave’ section of the Cool Kids and Chilled® Programs will be omitted for the purposes of this research as it was considered to have mindfulness components more consistent with an ACT approach. This is a small component of the overall program.

### ACT program - Cool Mind (ages 7 to 11 years) and Mindchill (ages 12 to 17 years)

The Cool Mind for Kids and Mindchill programs have been developed at CHW. They are both adaptations of the Cool Kids and Chilled® programs and were designed to conform to the overarching structure of these programs for comparison purposes. Cool Mind for Kids and Mindchill were developed on the basis of ACT-consistent protocols adapted from the Mindfulness-Based Cognitive Therapy for Children protocol [2]; Acceptance and Commitment Therapy Adapted for Children protocol [67], MiCBT protocol [68], and ACT Mindfully Workshops [69]. These programs incorporate all six ACT core therapeutic processes that target psychopathology including Acceptance, Being Present/Mindfulness, Valued directions, Committed Action, Self-as-context and Cognitive defusion. Children will learn skills to manage the distress associated with anxious thoughts and feelings. For example, whereas CBT attempts to dispute and modify unrealistic thoughts, ACT supports children to identify their values and behave in a value-consistent way. At the same time acceptance of anxious thoughts and feelings that may arise in the process of doing so is encouraged, as is learning to defuse these thoughts and feelings. While the content is similar for Cool Mind for Kids and Mindchill programs, the language is simplified for the child program and there is a greater focus on values for the adolescent program. Table 3 provides a session-by-session outline of the programs.

**Table 3:** Session-by-session overview of the Acceptance and Commitment Therapy programs, Cool Mind and Mindchill

**Session**	**Mindchill program (adolescents)**	**Cool Mind for Kids program (children)**
1	Introductions, group expectations and an emphasis on the importance of practice tasks; psychoeducation on anxiety, values and feelings; introduction to acceptance and mindfulness as alternatives to ‘getting rid’ of unpleasant thoughts and feelings. Practice tasks: Mindful smiling	Introductions, group rules, psychoeducation on anxiety, learning about feelings, Feeling/worry scale, pink elephant and Chinese finger trap exercises to introduce the futility of control and acceptance as an alternative; introduction to mindfulness. Practice tasks: What I think and feel, mindful smiling/breathing whilst waking up
2	Barriers to mindfulness practice, mindfulness of the breath, mindful eating, choosing to live a valued life regardless of fear, mindful movement, rewards, Practice tasks: Additional mindfulness practice	Mindfulness of the breath, mindful eating, thoughts/feelings and control; anxiety and my body, psychoeducation on acceptance; mindful movement. Practice tasks: Mindfulness of the breath, mindfulness while lying down, mindfulness in everyday activities, ‘Me and My Body’ physiological aspects of anxiety
3	Mindful breathing; ‘milk, milk, milk’ exercise as introduction to defusion; mindful thinking; acceptance versus tolerance of anxiety. Practice tasks: Defusion and mindfulness exercises	Mindful breathing; ‘milk, milk, milk’ exercise as an introduction to defusion; introduction to and practice of defusion strategies; mindful thinking; introduction to rewards. Practice tasks: Defusion exercise, mindfulness of the breath, mindfulness in everyday activities
4	Body scanning; creating a fears and worries list; mindful thinking practice; introduction to stepladders (exposure) and first attempt to create own exposure hierarchy. Practice tasks: Body scanning, mindful thinking and working on personal stepladder	Body scanning; mindful thinking practice; creating a fears and worries list; introduction to stepladders (exposure) and first attempt to create own exposure hierarchy. Practice tasks: Daily body scanning, mindful thinking worksheets, create first stepladder
5	Imaginal exposure using stepladders; experiential avoidance and taking our worries with us through life; mindful thinking additional practice; revising stepladders. Practice tasks: Mindfulness practice and stepladders	Imaginal exposure using stepladders; experiential avoidance and taking our worries with us through life; mindful thinking for big worries; stepladders for big worries. Practice tasks: Body scanning, mindful thinking and exposure
6	Mindfulness practice, leaves on a stream exercise; judging versus describing, ‘unhooking’ from thoughts; letting go of negative self-judgements, acceptance. Practice tasks: Stepladders	Mindfulness practice, leaves on a stream exercise; judging versus describing, acceptance; working on stepladders for big worries. Practice tasks: Mindfulness meditation, imaginal exposure practice about the worst that can happen if he or she confronts the difficult situation and work on stepladders
7	Body scanning additional practice; dealing with set-backs or getting stuck; coping strategies; problem-solving skills building. Practice tasks: Body scanning and imaginal exposure on stepladders	Body scanning additional practice; mindful touch; problem-solving skills building; mindful thinking. Practice tasks: Mindfulness body scanning, exposure (both imaginary and real life)
8	Mindfulness practice; assertive communication; in-session exposure related to stepladders or fears and worries list; problem-solving any set-backs. Practice tasks: Mindful thinking worksheets with stepladders, seeking feedback for areas of self-doubt	Mindfulness practice; assertive communication; in-session exposure related to stepladders or fears and worries list; problem-solving any set-backs. Practice tasks: Mindfulness thinking, exposure in real life, practicing problem-solving
9	Mindful breathing additional practice; coping with teasing and bullying; external strategies to manage worries; review of progress towards goals; additional in-session exposure. Practice tasks: Act on one goal not yet achieved, family discussion of managing anxiety and stress in everyday life	Mindful breathing additional practice; outsmarting bullies; review of progress towards goals; additional in-session exposure. Practice tasks: Practicing describing thoughts rather than judging, practicing assertiveness, mindfulness activities, 10 minutes/day
10	Loving kindness meditation; reviewing goals; planning for the future; dealing with set-backs and celebrating success	Friendly wishes meditation; reviewing goals; focus on values guiding action; planning for the future; dealing with set-backs and celebrating success

### Controls

The control group in the study will be a waitlist group, also diagnosed with an anxiety disorder. Participants allocated to this group will complete baseline assessments and will receive CBT following a waiting list period of 10 weeks. This period was selected as it corresponds with the 10 week program, allowing for comparative post measure. It was considered unethical to withhold treatment for longer than 10 weeks.

### Treatment fidelity

Across the group treatment period, all psychologists will receive weekly group supervision. As the psychologists involved in the delivery of the treatment are involved in the delivery of both the ACT and CBT treatments, treatment fidelity is an important consideration that will be addressed via video-recording a subset of each 10 week program. Video-recording will be conducted with the consent of all participants and will be reviewed and analyzed by an independent assessor, for fidelity with the identified treatment (ACT or CBT) in accordance with a checklist designed for this study. The independent assessor will be a psychologist, with training in the use of both ACT and CBT, within the Department of Psychological Medicine who is not involved in the treatment groups. Two independent assessors will be employed to ensure reliability of treatment fidelity ratings. Feedback on the outcome of these reviews will be provided back to the research team and subsequently explored within weekly supervision. In line with the protocol of Forman *et al*. [70], therapist allegiance towards treatment will be assessed by having each psychologist respond to the question ‘Which treatment do you think leads to better outcomes, ACT or CBT?’. Therapist allegiance will be examined as a variable of interest in terms of any possible association with study outcomes.

### Sample size calculation

A power analysis was conducted to determine the approximate sample size required to achieve an examination of the effectiveness of a manualized ACT group-therapy program, the most sensitive aim. At a power level of 0.8, with an effect size (ES) of 0.6 to 0.7, it is estimated that a minimum 30 participants in each group is required to detect a significant difference between each of the three groups. The employment of analysis of covariance and mixed model statistical tests for pre versus post comparisons will also offer increased power to detect significant differences. The estimated ES of 0.6 to 0.7 is based on the results of three previous meta-analyses of ACT. In terms of primary outcome measures, Hayes *et al*. [31] observed that, compared to structured interventions, ACT was superior after treatment (*d* = 0.48) and at follow-up (*d* = 0.63). Ost [71] found that ACT was superior to established treatments achieving a mean ES of 0.68. In contrast, Powers *et al*. [72] found that, on primary outcome measures, ACT achieved ES in the range *g* = 0.42 to 0.68 when ACT was compared with control or waitlist conditions and *g* = 0.18 compared to established treatments. The ES for the current study is estimated in line with the higher-end ES results achieved across the aforementioned meta-analyses. The rationale for this is that these meta-analyses have incorporated studies for a range of low-prevalence disorders - disorders that are typically considered to be treatment resistant - whereas the current study focuses only on anxiety, high prevalent disorders, widely acknowledged to be more responsive to treatment.

### Statistical analysis

Data coding and analysis will be conducted using the IBM SPSS Statistics v.21 software program. Primary outcome measures will be examined both with intention-to-treat analyses and analyses conducted on treatment completers. For the efficacy hypotheses, paired t-tests and linear mixed models will be used to investigate changes in pre versus post primary and secondary outcome measures. Statistical significance will be considered as *P <*0.05. The mixed model approach has been selected as it allows for inter-participant and intra-participant variance as well as the inclusion of participants with missing data, whilst maintaining power.

In accordance with the protocol of Forman *et al*. [70], mechanism of change hypotheses will be examined using correlation and independent t-tests to determine which variables to include in a multivariate analysis of variance. Post-hoc multiple comparisons (for example, Bonferroni correction) will be performed to determine where significant differences lie. Multivariate regression examining factors related to treatment success will also be employed and will include examining any demographic (such as age, gender, family functioning) and clinical (such as pre-scores, psychological flexibility) factors that might be related to outcomes. Mediational analysis, a regression-based approach, will be used to test hypotheses about the mechanisms behind outcome, as this is a powerful way of determining mechanisms by which an effect operates rather than the existence of an effect [73]. It allows for more than one mediator and adjusts all paths for the potential influence of covariates not proposed to be mediators in the model. Receiver operating curve plots of the true positive and false positive rate for different possible cut-points of the regression test will be conducted because receiver operating curves convey information relating to the trade-off between sensitivity and specificity, and maximize predictive value. The coordinates of the receiver operating curve will be used to determine the optimal cut-point for the test. The full information maximum likelihood method will be used to deal with missing data.

## Discussion

Whilst research has found that ACT is supportive in the treatment of anxiety in adult populations and has been found to be effective in the treatment of children with other concerns, there is a paucity of research examining the effectiveness of ACT among children with anxiety. To the best of the researchers knowledge, this study will be the first randomized controlled trial examining the effectiveness of ACT in young people with a diagnosed anxiety disorder. Conducted in a clinical practice context, this research will assess psychological interventions suitable for implementation in broader psychological settings. If found to be effective, this trial will support the development of ACT treatment protocols that could be made available to clinicians for use in both public and private contexts. It will offer the potential to provide an evidence base to support alternative treatment for anxiety in young people, particularly for those who do not benefit from standard treatments. This will allow for greater flexibility in the tools clinicians can use, and tailor treatment according to individual needs.

In terms of anticipated difficulties for the research, recruitment and retention of participants (particularly adolescents) has been identified as a potential caveat. A number of factors have been identified may contribute to this including the stringent inclusion and exclusion criteria, requiring a primary diagnosis of anxiety disorder and an absence of complex presentations such as suicidality; availability and interest of adolescents and caregivers to commit to attendance of at least 80% of sessions; and other issues specific to the population to be studied, such as School Certificate and Higher School Certificate examinations. To avert this anticipated concern, a proactive approach will be adopted to attempt to increase appropriate referrals to the Department of Psychological Medicine, CHW, and will include contacting current and potential referrers - pediatricians, general practitioners, psychiatrists, health professionals within child and adolescent mental health, and private practitioners – to inform them about the study. Direct referral to community members will be encouraged among completers of the programs; school newsletter publications and CHW’s Bandaged Bear Bulletin (distributed to all hospital staff and affiliates) will be employed to detail parents on the study; and physical advertisements will be placed in waiting rooms within CHW.

Other approaches will focus on school counselors, who often see adolescents with problems of anxiety, as previous research has found that schools were beneficial avenues for recruitment drives [74]. Methods of increasing referral rates will also include presentations of the research and inclusion criteria and peer networking undertaken by the researchers at various professional development courses as previous research with adolescents found that informing key decision makers and community sources in the early stages of the research were associated with better recruitment outcomes [74]. Another approach being considered is conducting adolescent groups at some of the high schools that have expressed interest. This will, however, require parents to attend in school time along with the students. Finally, other approaches will include cold calling and emailing to referrer mailing lists.

Although some studies have found monetary incentives facilitated increased recruitment and retention in research with adolescents [74], this has not been observed universally [75,76] and will not be feasible in the current study. Instead, engagement in the research will be supported by the provision of the treatment by registered psychologists experienced in the delivery of these programs at no cost and ensuring expectations of participants is communicated with clarity and consistency, particularly in the early stages of recruitment. Retention issues will also be addressed by enabling participants in the intervention groups to complete a ‘catch-up’ session the following week if they are unable to attend a session with one of the treating psychologists and encouragement of in-group socialization within the therapy to establish group cohesiveness. It is anticipated that these approaches will be somewhat supportive in addressing recruitment and retention issues in the current trial.

The researchers will publish outcomes of this trial in peer-reviewed clinical journals. Findings will also be disseminated at relevant conferences that would be attended by practicing clinicians. Findings will also be presented at CHW in-service days as well as for various community organizations in the Area Health Service, and school counselors can inform parents, increase community awareness and refer clinicians to the program.

### Trial status

Recruiting.

## Abbreviations

ACT: Acceptance and Commitment Therapy; ADIS-IV: Anxiety Disorders Interview Schedule for DSM-IV; AFQ-Y: Avoidance and Fusion Questionnaire - Youth; CALIS-C: Children’s Anxiety Life Interference Scale - Child Form; CAMM-20: Child and Adolescent Mindfulness Measure; CBCL: Child Behavior Checklist - Parent Form; CBT: Cognitive Behavior Therapy; CDI: Child Depression Inventory; CHW: The Children’s Hospital at Westmead; DSM-IV: Diagnostic Statistical Manual of Mental Disorders - Fourth edition; ES: effect size; FAD: Family Assessment Device; MASC: Multidimensional Anxiety Scale for Children; TAS-20: Toronto Alexithymia Scale; VLQ: Valued Living Questionnaire.

## Competing interests

The authors declare that they have no competing interests.

## Authors’ contributions

All authors contributed to the design of the study and assisted in the development of the protocol. KH gained ethical approval through CHW Human Research Ethics Committee and JS gained ethical approval through The University of Newcastle Human Research Ethics Committee. All authors contributed to manuscript preparation. All authors read and approved the final manuscript.
